# Transcutaneous vagal nerve simulation to reduce a systemic inflammatory response syndrome and the associated intestinal failure: study protocol of a prospective, two-armed, sham-controlled, double-blinded trial in healthy subjects (the NeuroSIRS-Study)

**DOI:** 10.1007/s00384-021-04034-1

**Published:** 2021-10-02

**Authors:** Cornelius J. van Beekum, Martin W. von Websky, Maria A. Willis, Christina Panknin, Martin Coenen, Rolf Fimmers, Jörg C. Kalff, Sven Wehner, Tim O. Vilz

**Affiliations:** 1 Department of General, Visceral, Thoracic and Vascular Surgery, University Hospital of Bonn, Bonn, Germany; 2 Clinical Study Core Unit, Institute of Clinical Chemistry and Clinical Pharmacology, University Hospital of Bonn, Bonn, Germany; 3 Clinical Study Core Unit, Institute of Medical Biometrics, Informatics and Epidemiology, Study Center, University Hospital of Bonn, Bonn, Germany

**Keywords:** Vagal nerve stimulation, SIRS, Inflammation, Intestinal barrier, Ileus, Cholinergic anti-inflammatory pathway

## Abstract

**Purpose:**

Surgery initiates pro-inflammatory mediator cascades leading to a variably pronounced sterile inflammation (SIRS). SIRS is associated with intestinal paralysis and breakdown of intestinal barrier and might result in abdominal sepsis. Technological progress led to the development of a neurostimulator for transcutaneous auricular vagal nerve stimulation (taVNS), which is associated with a decline in inflammatory parameters and peristalsis improvement in rodents and healthy subjects via activation of the cholinergic anti-inflammatory pathway. Therefore, taVNS might be a strategy for SIRS prophylaxis.

**Methods:**

The NeuroSIRS-Study is a prospective, randomized two-armed, sham-controlled, double-blind clinical trial. The study is registered at DRKS00016892 (09.07.2020). A controlled endotoxemia is used as a SIRS-mimicking model. 2 ng/kg bodyweight lipopolysaccharide (LPS) will be administered after taVNS or sham stimulation. The primary objective is a reduction of clinical symptoms of SIRS after taVNS compared to sham stimulation. Effects of taVNS on release of inflammatory cytokines, intestinal function, and vital parameters will be analyzed.

**Discussion:**

TaVNS is well-tolerated, with little to no side effects. Despite not fully mimicking postoperative inflammation, LPS challenge is the most used experimental tool to imitate SIRS and offers standardization and reproducibility. The restriction to healthy male volunteers exerts a certain bias limiting generalizability to the surgical population. Still, this pilot study aims to give first insights into taVNS as a prophylactic treatment in postoperative inflammation to pave the way for further clinical trials in patients at risk for SIRS. This would have major implications for future therapeutic approaches.

## Introduction

Trauma or major surgery (cardiac surgery, abdominal surgery, organ transplantation) results in liberation of pro-inflammatory cytokines as well as recruitment and migration of leukocytes. This can lead to hypotension and capillary leakage followed by a systemic inflammatory response and multiorgan dysfunction syndrome (SIRS and MODS) [1–3]. Hypotension and inflammation can diminish gastrointestinal peristalsis, resulting in a paralysis clinically known as “septic ileus” [4]. In consequence of the intestinal stasis, bacterial overgrowth occurs. Simultaneously, a breakdown of the epithelial barrier function due to ischemia allows translocation of enteral bacteria into the bowel wall or even beyond this line, with SIRS and abdominal sepsis as the most severe consequence, respectively [4]. Dysfunction of the gut is therefore considered a major prognostic factor in intensive care medicine as “the gut is the motor of critical illness” [5, 6]. Until now, neither a prophylaxis nor targeted therapy for SIRS exist. The treatment is symptomatic and includes resuscitation catecholamine treatment and organ replacement in case of MODS. Costs of a prolonged stay on the intensive care unit due to complications of SIRS and bowel dysfunction exceed 25.000 USD per patient [7, 8], highlighting the importance of research in prevention and therapy of SIRS.

Since the late 1990s, it is known that the vagal nerve (VN) plays an important role in immunological homeostasis. A group led by Kevin Tracey demonstrated that a transection of the VN at the cervical level led to a higher mortality after lipopolysaccharide (LPS) challenge in rodents. Electrical stimulation of the distal end of the severed VN (VNS) resulted in a lower systemic inflammatory response and improved survival after the LPS challenge. In the manuscript, the so-called “Cholinergic Anti-inflammatory Pathway” (CAIP) was described for the first time [9]. More experiments using animal models of SIRS or sepsis were able to show that activation of the CAIP with electrical VNS led to a significantly reduced intestinal and systemic inflammation with a more pronounced peristalsis and shortening of duration of motility disorders and SIRS [10–14]. Furthermore, VNS improved stability of intestinal tight junctions during experimental sepsis, leading to a stronger intestinal barrier function with less bacterial translocation and a reduced mortality in a rodent model [15].

These animal studies led to the idea that electrical VNS after surgical implantation of a stimulation electrode (invasive VNS (iVNS)) proximal to the cervical VN could be an ideal treatment for inflammatory diseases. In a human pilot trial in patients with rheumatoid arthritis, a significant decrease in TNF-α concentration, as well as a reduction of symptoms, was observed after iVNS. In patients with therapy refractory Crohn’s disease, a reduction of Crohn’s activity index was observed [16, 17]. However, no randomized controlled trial could demonstrate a convincing and permanent effect of iVNS in chronic inflammatory diseases. This is possibly due to the permanently smoldering inflammation caused by the autoimmune disease, which cause cannot effectively be treated by activation of the CAIP.

IVNS has some major drawbacks: A surgical procedure with exposure of the cervical VN is mandatory to implant the stimulation electrode which could lead to vascular and nerval injury due to the close relationship of VN and carotid artery or jugular vein. Furthermore, a prophylactic stimulation before the beginning of an acute inflammatory stimulus, i.e., before major abdominal surgery, would be ethically difficult to justify due to an increased risk of side effects by the additional invasive procedure.

To overcome these limitations, new strategies aimed to identify non-invasive options of VNS to activate the efferent arm the CAIP. One opportunity is the stimulation of afferent fibers of the auricular branch (auricular branch of the vagal nerve, ABVN) at the concha auricularis [18]. It has been shown that a non-invasive VNS at the concha activates brain centers, particularly the nucleus tractus solitarii (NTS), what finally results in a circuitry of the electrical signals into the efferent vagal fibers targeting and innervating peripheral organs including the lung, the heart, and the gastrointestinal tract [19–21]. Interestingly, this transcutaneous auricular VNS (taVNS) activates the same regions in the brain as it has been shown for iVNS before [22]. Several studies investigated taVNS in treatment of depression, epilepsy, migraine, chronic pain, and atrial fibrillation, displaying its beneficial properties [23–26]. In other trials, it was shown that taVNS can improve gastroduodenal motility, accelerate gastric emptying, and relieve symptoms of functional dyspepsia [27–29]. However, until today, immunomodulatory effects of taVNS have rarely been investigated. First evidence for a peripheral immune modulatory action after taVNS came from a previous rodent study of our group. In a mouse model of paralytic ileus induced by surgical manipulation of the small bowel, we demonstrated that inflammation of the muscularis externa as well as bowel dysmotility was diminished by perioperative taVNS [14]. In a following pilot study in humans undergoing open abdominal surgery, we confirmed that intraoperative taVNS indeed increased peripheral smooth muscle activity of the stomach as visualized by an increase in frequency and amplitude of action potentials of the muscularis. Furthermore, increased serum levels of gastrin, serving as a surrogate marker for activation of the VN, were detected postoperatively [30]. Interestingly, a decline of inflammatory cytokines such as TNF-α in sera of patients treated with taVNS for depression, atrial fibrillation, and myocardial infarction has also been observed [25, 31–33]. However, these patients were not confronted with an inflammatory stimulus before VNS. A human trial investigating the effects of taVNS during acute inflammatory stimuli is lacking. In summary, existing promising data indicate that the electrical signals applied by taVNS to an afferent VN branch can indeed undergo circuitry to the efferent VN parts and therefore should also be able to activate the CAIP. Therefore, we hypothesized that taVNS can improve clinical symptoms of SIRS by dampening the systemic immune responses and prevention of gastrointestinal failure.

An ideal indication of taVNS could be a prophylactic treatment before and during major surgery to prevent the development of postoperative SIRS followed by gastrointestinal failure and MODS. However, the development of SIRS is multifactorial and depends on the extend of surgery, volume of transfusion and other factors. As predictive markers do not exist, surgeons cannot identify patients who might suffer from SIRS in principal and how severe the symptoms might be. Therefore, a clinical trial in surgical patients would require a large patient cohort what in turn is associated with a high economic burden. As a proof-of-concept study, a standardized study in healthy probands subjected to a controlled condition mimicking the inflammatory response during SIRS would be a more defined starting point, allowing investigation of the hypothesized prophylactic effects of taVNS on systemic inflammation.

**Fig. 1:**
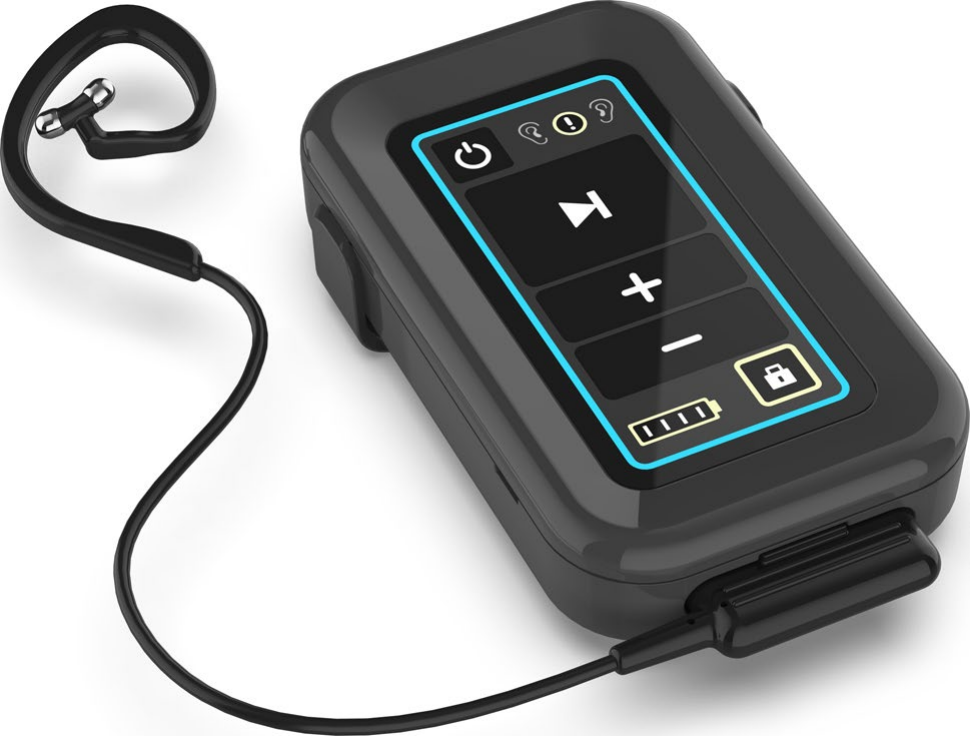
The tVNS®L-device for taVNS, provided by tVNS Technologies GmbH, Germany

**Fig. 2:**
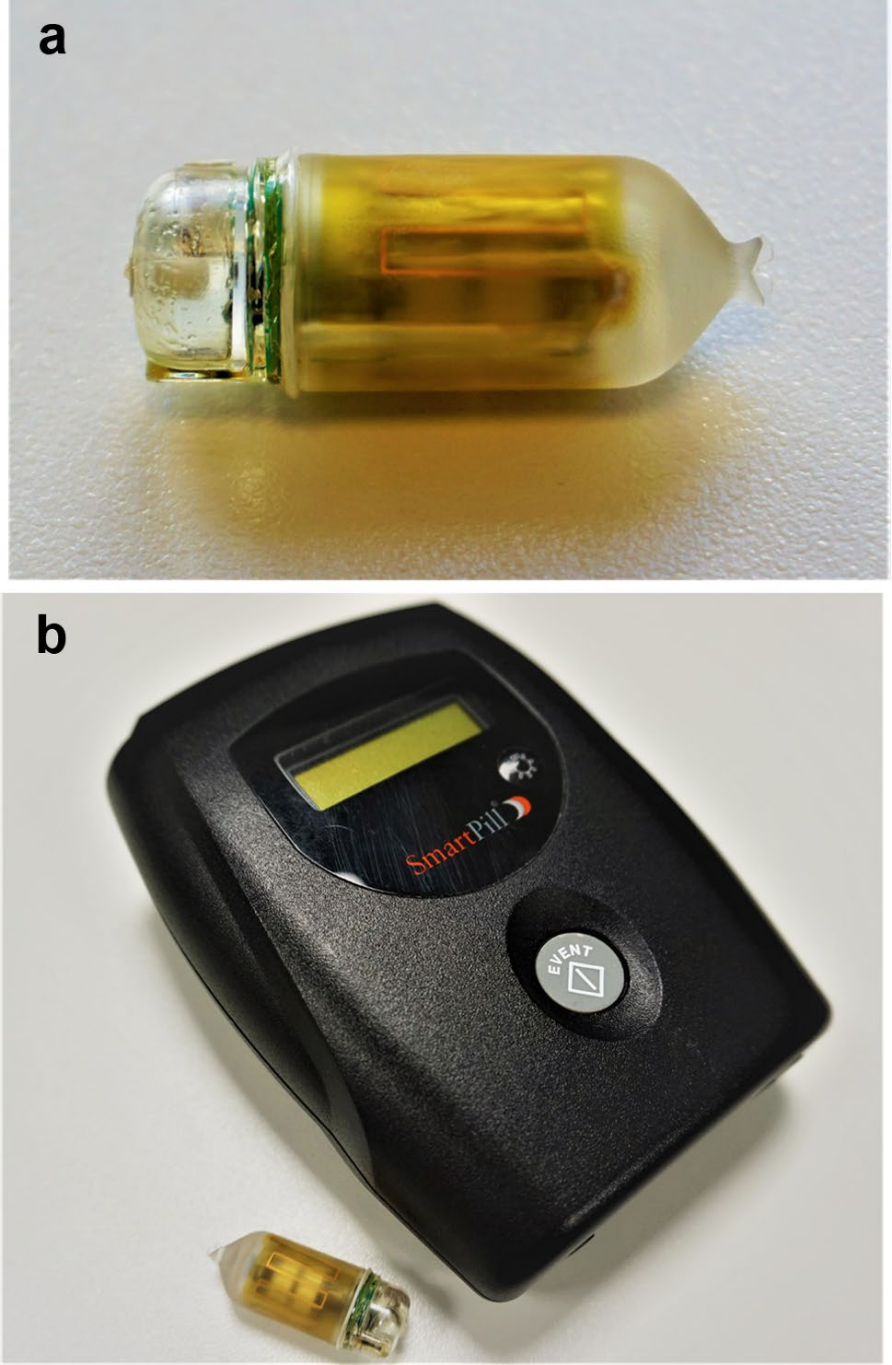
**a** The SmartPill®, a capsule sized 26 × 13 mm, measuring temperature, intraluminal pressure, and pH value in the intestinal tract. **b** The SmartPill® with the transportable receiver

We therefore designed a prospective-randomized, two-armed, double-blind clinical trial investigating the impact of taVNS in healthy subjects during a standardized model of experimental systemic inflammation. For taVNS, we use the tVNS®L device (Fig. 1) allowing an in-ear stimulation of the afferent VN fibers (or a sham stimulation in the control group, i.e., application of the device without starting stimulation). Standardized inflammation will be induced using the well-established human LPS model described by Fullerton et al. [34]. Inflammatory and anti-inflammatory blood parameters as well as clinical symptoms and gastrointestinal motility will be examined. The latter will be investigated by use of the SmartPill® (Fig. 2a) which allows an objective analysis of gastric emptying, small bowel, and large bowel transit times as well as peristaltic activity in every part of the GI tract [35, 36].

## Methods and analysis

### Sample size, recruitment, and blinding

The NeuroSIRS-Study is a prospective, randomized, two-armed, sham-controlled, double-blind, exploratory clinical trial in healthy subjects. It is a pilot study with the intention to gain first insights into the primary and secondary objectives. Therefore, sample size is not based on a statistical rationale. Thirty participants will be enrolled in the study: 15 subjects in the experimental group and 15 subjects in the control group. Healthy volunteers will be recruited by advertisements placed at the University Hospital of Bonn and in the local newspaper. During the screening visit, potential participants will be informed about the NeuroSIRS-Study. After sufficient time for consideration, a written informed consent will be obtained. Participants failing to meet inclusion criteria will not be enrolled in the study.

Since the NeuroSIRS-Study is an exploratory trial designed to give first insight into the impact on taVNS during systemic inflammation and to achieve the best possible comparability, it was decided to include only Caucasian men into the study. To ensure equal distribution of known and unknown variables between both groups, a randomization will be carried out upon enrolment. Patients are randomized to one of two therapy arms (arm A: taVNS application; arm B: sham stimulation/control). Randomization will be performed using central fax randomization according to a randomization list supplied by the Institute of Medical Biometrics, Informatics and Epidemiology, Study Center, University Hospital of Bonn. Participants and study personnel will be blinded until completion of the NeuroSIRS-Study. Application of the taVNS device will be carried out by study personnel. Stimulation settings (electrical stimulation or sham, i.e., no stimulation) will be adjusted by an independent physician who is otherwise not involved in the study. The stimulation settings will be concealed without the possibility for the investigator or the participant to see the settings.

### Data collection and management, monitoring, safety management, confidentiality

The Clinical Study Core Unit of the Study Center Bonn (CSSC) will perform the monitoring, safety management, data management, and data analysis of the study. All data relating to study participants will be stored on a secured and encrypted server only accessed by the investigators. Subjects will be assigned by alphanumeric sequential numbers that will be used to identify clinical data. On completion of the study, all participant-identifying information and other study data will be securely archived according to the applicable laws and regulations. The case report forms only include pseudonymized data of the study subjects, and only qualified persons will have access. Personal information of the study subjects will be stored in the ISF at trial site. Only qualified persons who are contributing to the clinical trial have access to these data. All trial data are collected pseudonymously.

Safety management will be in the responsibility of the CSSC and will be performed according to established SOPs. Adverse events will be documented by the staff of the trial site according to the requirements of the ICH-GCP and the ISO14155 guideline following enrolment of the subject into the trial. A safety management plan describing the trial-specific procedures was compiled. Safety data collection, documentation, and reporting of adverse events will be performed according to the applicable laws and regulations (German Medical Device Law, MPSV, EU-directive 2007/47/EG, DIN EN ISO 14155:2012, MEDDEV 2.7/3, Declaration of Helsinki).

There are no plans for systematic auditing by the sponsor. Depending on the course of the trial, triggered audits are possible any time. Owing to the German legislation concerning medical devices, the NeuroSIRS-Study will be inspected by the district government. The government reserves the right for further auditions depending on the occurrence of serious adverse event (SAE) or serious adverse device effects (SADEs). The process will be independent from the sponsor.

### Objectives

This study aims to investigate whether symptoms and consequences of standardized systemic inflammation can be influenced by taVNS.

#### Primary objectives

The primary objective of the study is to investigate whether clinical signs of LPS-induced SIRS can be influenced by taVNS.

#### Secondary objectives

1. Evaluation whether taVNS can influence gastrointestinal motility during SIRS by comparison of intestinal pH, intraluminal pressure, and peristaltic activity between both groups. Intestinal barrier function will be compared using the lactulose/mannitol ratio between the two arms.
2. Change of activity of the autonomous nerve system is investigated during and after taVNS or sham stimulation via heart rate, blood pressure, and heart rate variability.
3. Safety of taVNS after LPS application is evaluated based on the rate of adverse events (AEs), serious AEs, adverse device-related events (ADEs), and serious ADEs.
4. Affection of the immune system by comparison of the plasma concentration of pro- and anti-inflammatory cytokines such as TNF-α, IL 6, IL 8, IL 10, IL 18, IL 1β, and HMGB-1 as well as immune cells before and after taVNS or sham stimulation.

### Inclusion criteria

Written informed consent, age > 18 and < 45 years, male gender, Caucasian ethnicity.

### Exclusion criteria

Age < 18 years; > 45 years; female sex; active smoker; constipation or diarrhea; surgical procedures or trauma-necessitating blood transfusion; donation of blood within the past 4 weeks; immunosuppressive or immunomodulatory medication in the past 6 months; medication with known influence on cholinergic, dopaminergic, adrenergic, or serotoninergic neurotransmission; history of cardiovascular disease or vasovagal/orthostatic syncope in the past 12 months; heart rate < 45/min or > 110/min; systolic blood pressure > 160 or < 90 mmHg; known polarization and repolarization disturbances; obstructive pulmonary disease or allergic asthma; pacemaker or internal defibrillator or any other electrical implant; seizures; impaired kidney function with serum creatinine above average; alkaline phosphatase or aspartate transaminase three times higher than normal; immunosuppression (hereditary, acquired, or drug induced); c-reactive protein above average and/or leukocytosis above average or current infection; infection within past 2 weeks before study; known allergy or hypersensitivity to one of the components of the medical device; conditions or diseases which do not fit with the study at the investigator’s discretion; known dysphagia; non-steroidal anti-inflammatory drug enteropathy in the past; patients with cerebral shunts; patients with active implantable devices, medication with proton pump inhibitors (PPIs), antacids, or H2 blockers; reflux esophagitis grade III and IV according to Savary and Miller; fistulas of the esophagus and/or stomach; known or suspected stenoses or fistulas of the GI tract; active Crohn’s disease; diverticulosis or diverticulitis; any abdominal surgery in the past 3 months; history of abdominal surgery (excluded: appendectomy, cholecystectomy, or any minimally invasive performed surgery); type I diabetes; diseases of the adrenal glands; splenectomy; prior participation at a study with LPS application; incapacity to fully understand the study; known misuse of alcohol, medication, or drugs in the past; former participation in LPS challenge.

### Intervention

After completed screening (medical history, physical examination with ECG, blood pressure, O2 saturation, and blood tests), participants ingest the SmartPill® and leave the hospital. The capsule analyzes peristaltic activity as well as gastric emptying time, small bowel transit, large bowel transit, and whole gut transit in the absence of an inflammatory stimulus. Those measurements serve as an intraindividual reference, as transit time and motility vary between individuals. After 5 days, subjects return to the phase I unit of the hospital to proceed with the trial. Upon confirmation of excretion of the capsule, another SmartPill® is ingested.

Subjects are randomized and either undergo taVNS or sham stimulation for 30 min before LPS application. LPS is administered at 2 ng/kg bodyweight to all subjects. One hour after administration, taVNS is terminated. ECG and SpO2 are measured continuously for 3.5 h, starting just before stimulation. Blood pressure, body temperature, and respiratory rate are measured every half hour for 3 h. Until discharge of the participant, vital signs are measured hourly.

LPS‐induced flu‐like symptoms (headache, nausea, shivering, muscle, and back pain) are scored per symptom (0 = no symptoms, 5 = worst ever experienced, vomiting: additional 3 points resulting in a total symptom score of 0–28) before taVNS and at 1 h, 1.5 h, 2 h, 2.5 h, 3 h, 3.5 h, 4 h, 5 h, 6 h, and 7 h after the beginning of taVNS as suggested by Kox et al. [37–40].

During screening; before taVNS; before starting of LPS administration; at 1 h, 1,5 h, 2 h, 2.5 h, 3 h, 4 h, 6 h, and 7 h after the beginning of taVNS; and at the final examination blood samples are drawn and analyzed for pro- and anti-inflammatory parameters (TNF-α, IL 6, IL 8, IL 10, IL 18, IL 1β, HMGB-1) and changes in blood cell count.

Subjects leave the hospital and return after 5 days to confirm capsule excretion and for final clinical examination (Table 1).

**Table 1:**
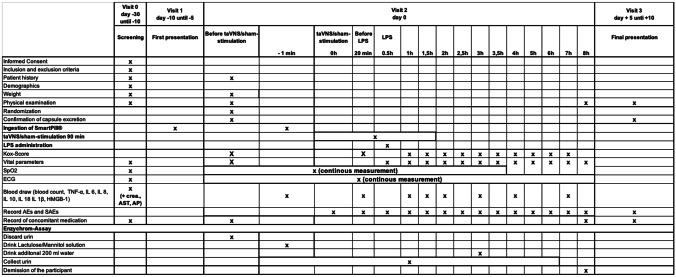
Study schedule

*taVNS* transcutaneous auricular vagal nerve stimulation, *LPS* lipopolysaccharide, *SpO2* peripheral oxygen saturation, *ECG* electrocardiogram, *(S)AE* (serious) adverse event

#### LPS

LPS (*Escherichia coli* O113) is obtained from List Biological Labs, Inc., Campbell, CA, USA. LPS (2000 EU/ml) will be subjected to a microbial safety testing routine and confirmation of concentration as recommended by the German Federal Agency for Sera and Vaccines (Paul-Ehrlich Institute, Langen, Germany). LPS will be stored in endotoxin-free borosilicate tubes at –20 °C until use.

#### taVNS device

The tVNS®L device (Fig. 1) for taVN stimulation is provided by tVNS Technologies GmbH, Germany. Stimulation will be performed at 25 Hz, placing the auricular electrode upright in the left external ear. The left ear is chosen to avoid cardiac side effects and to increase outflow to the spleen, which is implicated in mediating the anti-inflammatory effects of VNS [37–39]. Intensity of stimulation will be increased until the participant describes a “tingling” sensation in the ear, without the sensation of pain. Usually, this tingling sensation is reached at 0.3–0.8 mA.

#### Analysis of intestinal function

##### Investigation of peristaltic activity and transit times

The SmartPill® is a capsule sized 26 × 13 mm (Fig. 2a) and was developed for analysis of bowel motility [35, 36]. The device is ingested measuring temperature, intraluminal pressure, and pH value in the intestinal tract. The acquired data is sent and stored within a transportable receiver (Fig. 2b), which allows continuous monitoring and analysis of the data after capsule excretion using the MotiliGI^©^ software. By analyzing these parameters, gastric emptying time (increasing temperature after capsule ingestion until sudden rise of the pH value when leaving the stomach and small bowel transit (sudden pH increase after entering the duodenum until an increase of pH value for one point and changes in the pressure profile when passing Bauhin’s valve) as well as large bowel transit (passage of Bauhin’s valve until drop of temperature when the capsule enters the toilet water) can be determined. Furthermore, intensity (intraluminal pressure) and frequency of peristaltic activity (contractions per minute) in all sections of the intestinal tract can be detected.

##### Investigation of intestinal barrier function

Viability of participant´s intestinal barrier function during the LPS challenge and taVNS will be assessed using the EnzyChrom Intestinal Permeability Assay Kit. Participants will be asked to empty their bladder and drink 200 ml of water containing 10 g of lactulose and 5 g of mannitol. Urine will be collected for 6 h thereafter. After 3 h, participants will drink an additional 200 ml of water. No additional fluid will be applied before or afterwards. A sample of the participant’s urine will be taken, and concentrations of the excreted sugars will be analyzed. Calculating the ratio of excreted lactulose and mannitol, integrity of intestinal tight junctions can be investigated.

#### Experimental group

Participants will be stimulated with 20 Hz transcutaneous at the concha of the left outer ear for 30 min, before LPS is administered at 2 ng/kg bodyweight under constant control of vital parameters. Stimulation will be continued for 60 min after LPS application.

#### Control group

taVNS electrodes are placed in the ear, but stimulation will not be started (sham stimulation). LPS will be administered the same way.

### Safety aspects

The NeuroSIRS-Study will be conducted at the Phase I, Clinical Study Core Unit at the University Hospital of Bonn, Germany. Here, participants will be connected to a monitoring system, measuring heart rate (electrocardiogram, ECG), blood pressure (in intervals, non-invasively), oxygenation and respiratory rate continuously for 3.5 h. Body temperature will be taken upon arrival and after lying down for 15 min. For analysis of heart rate variability as a marker for sufficient VNS, ECG will be taken until dismissal from the Phase I unit.

Safety of taVNS during systemic inflammation will be assessed based on the rate of AEs, SAEs, ADEs, and SADEs.

### Statistical analysis

The analysis of the study data will be descriptive. Differences for primary and secondary outcome parameters between the treatment groups will be estimated with 95% confidence limits. The evaluation of the effectiveness parameters will be performed with all randomized subjects first and in addition for the subjects who were compliant to the protocol. The evaluation of the safety parameters is carried out with all subjects on whom one of the study procedures was carried out in whole or in part.

Study participants who withdraw from the study prematurely before the second application of SmartPill® will be replaced by additional study participants. Likewise, study participants who have a malfunction of the SmartPill® will be replaced. The probability of a malfunction of the capsule is estimated by the distributing company to be less than 5%.

All test persons who leave the study prematurely will be listed and monitored. A follow-up is not necessary. The reason for their withdrawal is documented. The data available up to the time of withdrawal are included in the evaluation. Individual missing values are not replaced for the evaluation.

## Discussion

Trauma or major surgery go along with initiation of pro-inflammatory cascades leading to a variably pronounced SIRS [41]. Severity of SIRS (next to patient-specific variables) depends on the type, site, invasiveness, and duration of the performed procedure with an incidence of up to 60% after major surgery [42]. Inflammatory intestinal dysfunction, characterized by gastrointestinal motility disorder and a breakdown of intestinal barrier, appears to be a major prognostic factor. No preventive or directed treatment exists [5, 6].

Beside the pioneering work of Tracey et al., multiple other studies in rodents demonstrated the beneficial effects of CAIP activation via VNS before LPS challenge in the last years. For example, Zhou and co-workers showed a reduced cytokine release and a prevention of intestinal tight junction breakdown with a reduced bacterial translocation after LPS application and VNS. Our group as well as Huston and co-workers demonstrated beneficial effects of VNS in rodent LPS models with reduced cytokine liberation and an improved survival [15, 30]. In humans, there is growing evidence that a reduced vagal tone is associated with an increased morbidity and mortality after surgery-related SIRS or sepsis [43, 44]. It seems obvious that prophylactic stimulation of the vagal tone and thereby the CAIP might mitigate postsurgical morbidity related to postoperative inflammation. However, clinical trials investigating the prophylactic effect of VNS in acute postoperative inflammation in humans are lacking. As the occurrence and severity of postoperative SIRS vary greatly depending on various factors and cannot be predicted, a randomized controlled trial investigating SIRS prophylaxis using VNS requires a large sample size with an associated high financial burden. To avoid the required large sample size due to unpredictable SIRS occurrence, a reproducible and standardized model mimicking a systemic inflammation as it occurs during SIRS was expected to be helpful for a pilot trial. We therefore established the present study protocol to investigate the potential prophylactic effects of taVNS in a model of systemic inflammation.

### LPS challenge to induce a standardized systemic inflammation

To induce a standardized and reproducible SIRS, LPS challenge will be performed. The use of LPS to cause experimental systemic inflammation has been extensively investigated, showing no severe or long-term adverse effects [34]. Administration of LPS at a dosage of 2 ng/kg bodyweight induces specific physiologic and metabolic processes comparable to SIRS with culmination of symptoms after approximately 2 h, making it an ideal model to investigate potential prophylactic or therapeutic tools in systemic inflammation [45]. In the literature, dosing of LPS varies between 0.2 and 4 ng/kg bodyweight in human studies. We decided to administer LPS at this dosage since it is the most used and has been shown to induce distinguished symptoms of systemic inflammation with only minimal and tolerable side effects. The crucial difference to actual SIRS lies in the complete reversibility of symptoms after 6–8 h and the absence of secondary health damages [46–48].

Of note, LPS challenge leads to symptoms of SIRS, which might cause greater discomfort to individual participants. If subjects suffer from these symptoms during the trial, paracetamol can be administered at any given time, ameliorating these symptoms. In these participants, a potential interaction with the systemic inflammatory response needs to be considered during later analysis.

To achieve the highest level of safety, all participants are monitored with an ECG, blood pressure and peripheral blood oxygenation during the trial. Of course, proper pre-interventional information, consent of the participants, a thorough examination, and history-taking are mandatory before inclusion into the study. To evaluate SIRS-related symptoms and the inflammatory extent of LPS administration, a clinical score in conjunction with vital parameters as well as inflammatory cytokines in patient’s sera will be used. This triad of variables has been used in a plethora of clinical trials and is well-validated as a diagnostic tool to describe LPS-related SIRS [37–39]. A potential amelioration of the inflammatory extent of LPS administration through CAIP activation via VNS can be displayed.

### taVNS for activation of the CAIP

Electrical stimulation of the VN is usually accomplished by implanting a stimulation electrode near the cervical VN. However, to use VNS as a prophylaxis, a surgical procedure under general anesthesia before SIRS inducing surgery would be mandatory to implant the stimulation electrode in proximity to the cervical neurovascular bundle (carotid artery, jugular vein, VN). Furthermore, the electrode as well as the stimulator needs to be explanted after its use, necessitating another procedure. Considering possible surgical complications associated with electrode implantation and explantation, treatment of postoperative SIRS using iVNS seems infeasible.

Kox and colleagues thought of a model of cervical VNS using a special stimulation catheter placed in the jugular vein near the VN. The idea behind the intravenous stimulation is extremely interesting because during more invasive surgical procedures patients usually require a central venous line anyway. Therefore, using the transvenous stimulation catheter, prophylactic stimulation would be possible without exerting unnecessary harm to patients beyond a cervical venous puncture. In the trial, after placement of the stimulation catheter and VNS, participants underwent LPS challenge. Surprisingly, no anti-inflammatory effects of jugular VNS in terms of reduction of symptoms or cytokines in participants sera were seen [39]. The actual reason behind the missing anti-inflammatory effect of transvenous VNS is not yet understood. It is arguable that only efferent fibers of the VN were activated and that the stimulation was not strong enough possibly reducing a potential anti-inflammatory effect. Kox and colleagues later argued that different ways of VNS need to be investigated with emphasis on non-invasive auricular VNS with stimulation of afferent vagal fibers necessitating central nervous interconnection, potentially enhancing anti-inflammatory effects [37].

Technical progress within the last years led to the development of a device for taVNS. Interestingly, functional MRI studies could verify that taVNS via ABVN led to an activation of the same brain areas as iVNS suggesting comparable effects [22]. Avoiding risks associated with surgical implantation of a stimulation electrode for iVNS and taVNS allows for non-invasive stimulation of the VN. The afferent branch of the VN innervating the concha auricularis (ABVN) is stimulated transcutaneously with electrical impulses applied via electrodes connected to a handheld stimulation device sized comparable to a smartphone (Fig. 1). The ABVN then projects to the NTS leading to activation of a complex neuronal network lastly resulting in activation of the dorsal motor nucleus [22]. As demonstrated before, stimulation will commence at 0.1 mA and will be increased until the participant perceives a tingling sensation. Further increase of intensity will define the threshold when the sensation becomes discomforting. The intensity will stay below this threshold. The optimal intensity varies between 0.3 and 0.8 mA [21, 49, 50]. Recent research has shown that setting the stimulation threshold just below the verge of a tingling sensation, becoming painful at the outer ear, is strongly correlated with activation of the vagal nerve, as shown, for instance, in changes in heart rate variability [51]. It currently is the most standardized and reproducible modus operandi in taVNS. It is hypothesized that a tingling sensation is necessary because taVNS below a painful threshold should recruit myelinated auricular Aβ fibers responsible for cutaneous touch sensation and fibers responsible for central activation of vagal nuclei instead of myelinated Aδ fibers for cutaneous nociception [52].

Several trials were able to show that not only iVNS dampens inflammatory reactions, but also taVNS leads to decreased serum levels of IL 1β, IL 6, TNF-α, and HMGB-1 in patients with myocardial infarction or atrial fibrillation compared to sham-stimulated controls. These results indicate a close relation of vagal tone and immune response [25, 31, 32, 53]. However, there are no existing trials investigating the prophylactic use of taVNS before activation of an inflammatory cascade. Considering the interesting results of CAIP activation in animal models, a prophylactic stimulation before beginning of an inflammatory stimulus appears to be the key to successful abrogation of inflammation [14, 54]. This finding is supported by a recent study of Kox and colleagues demonstrating the efficacy of the acetylcholine receptor agonist and CAIP activator GTS-21 antecedent to LPS stimulation leading to a decrease in pro-inflammatory cytokines in healthy subjects [38]. Irrespective of the catalysator of the inflammatory reaction (surgery, trauma) and its associated risks, taVNS allows for harmless CAIP activation at any time, even in a preventive setting [55].

A great advantage of taVNS is that only few and minor side effects have been described and they mostly occur only during long term stimulation. For example, in the treatment of epilepsy, it was rarely associated with local side effects at the site of stimulation like itching, dysesthesias, rash, and other skin-related symptoms. Single cases of interference with the autonomic nervous system like headache, nausea, vertigo, bradycardia, or bronchoconstriction have also been described [56–59]. To ensure highest safety levels, the NeuroSIRS-Study will be performed at the Phase I trial unit of the University Hospital of Bonn, with participants being under continuous monitoring and always supervised by a physician. In case of unexpected severe side effect and emergency, a team of experienced physicians of the intensive care unit are readily available.

To date, only one exploratory clinical trial was able to show anti-inflammatory properties of perioperative taVNS in patients undergoing thoracic surgery showing a decline in IL 6 levels and infectious complications postoperatively. Putative intestinal effects were not studied [60]. In addition to this, Chapman and colleagues recently demonstrated in a pilot study with a small case number a reduced time to first flatus and faster solid food tolerance after colorectal surgery preceded by transcutaneous cervical VNS (stimulation of the efferent branches of the VN, no stimulation of the ABVN) compared to a sham-stimulated control group. The trial firstly described a motility-enhancing effect of non-invasive VNS postoperatively; unfortunately, inflammatory markers were not investigated [61].

Considering data from animal models showing the anti-inflammatory and motility-enhancing effects of taVNS, current knowledge about the association of vagal tone and outcome after SIRS and first trials investigating taVNS during lung or colorectal surgery, a clinical trial to investigate taVNS as a prophylactic treatment for SIRS and SIRS-related disruption of intestinal viability seems to be mandatory and consequent.

### Assessment of intestinal motility and barrier integrity

Intestinal viability will be evaluated using a composite analysis of intestinal motility and integrity of intestinal barrier function. At first, intestinal motility during systemic inflammation will be investigated using the SmartPill®. The SmartPill® has already been widely used to assess intestinal peristalsis in patients with impaired intestinal motility such as diabetic gastropathy or chronic constipation. We recently conducted a clinical trial demonstrating the safety of the SmartPill® in patients undergoing abdominal surgery, acquiring first objectifiable data on postoperative ileus [35]. To our knowledge, there is no parameter or biomarker to measure impairment of intestinal motility during SIRS-associated paralytic ileus. As the reduced perfusion followed by lack of peristalsis is the initial step of intestinal failure which finally leads to bacterial overgrowth and translocation, we hypothesize that the SmartPill® will be of great value to deliver objectifiable, reproducible data concerning the impairment of gastrointestinal motility in our standardized SIRS model. Lack of peristalsis in combination with impaired intestinal perfusion due to hypotension results in stasis, bacterial overgrowth, and finally the breakdown of intestinal barrier function with bacterial translocation and abdominal sepsis. This pathophysiological cascade led to the idea that the gut is the motor of critical illness [6]. Therefore, improving not only peristalsis but also the maintenance of barrier function would be a crucial step in SIRS prophylaxis. As it was shown in a rodent model, taVNS is able to prevent LPS-induced intestinal barrier breakdown resulting in a diminished bacterial translocation [15].

Integrity of the intestinal barrier will be investigated via analysis of the lactulose/mannitol ratio in participant’s urine. Urinary excretion of the two orally administered non-metabolizable sugars, lactulose and mannitol, is a valuable marker for evaluating intestinal permeability. While mannitol, a sugar monomer, is absorbed trans-cellular, lactulose, a dimer, is absorbed to a lesser extent and therefore marks mucosal integrity. We chose this model due to its non-invasive, easy-to-use nature and, to our knowledge, no known interactions between the sugars and the immune response. The test has been used in a variety of different indications and is a sensitive parameter to assess intestinal health [62].

### Limitations of the NeuroSIRS-Study

Our study offers several opportunities but has also some limitations. Small sample size might be considered as a possible limitation of the NeuroSIRS-Study. Nevertheless, it is a pilot study and designed to acquire first data concerning the influence of prophylactic taVNS on systemic inflammation in a highly standardized model. To induce systemic inflammation, LPS challenge is used. Notably, intravenous administration of LPS only leads to activation of a single toll-like receptor, while postoperative SIRS originates from a variety of inflammatory stimuli [34]. It might be argued that LPS challenge only partially mimics postoperative inflammation and is an artificial model not reflecting clinical reality. Even so, the LPS challenge remains the most used, ethically acceptable, and well-established strategy to systematically investigate SIRS and potential treatments in a standardized and reproducible manner.

Another crucial point is the side effects associated with LPS administration and dosage. Errors in dosing and dilution must be avoided to prevent severe side effects and potential harm to participants. Usage of LPS has been approved by the local ethics committee as it is the most standardized and safest way to induce systemic inflammation in an experimental setting. Nevertheless, even at a dosage of 2 ng/kg bodyweight, potent symptoms of SIRS are to be expected. Comparable to a “flu-like” infection, body temperature is expected rise above 38 °C; shivering and fatigue may occur. Hypothetically, allergic reactions towards LPS are possible but rather unlikely considering that the substance is highly purified and processed under “good manufacturing process” conditions by a certified institution. Since individual case reports on LPS-induced bradycardia exist, patients with a history of vagal reactions or pre-existent bradycardia are excluded from the study [63, 64]. If symptoms of inflammation are experienced strongly, paracetamol can be administered to weaken these symptoms. Consecutively, recruitment, an issue in every clinical trial, could be challenging due to the likely side effects. However, to guarantee the highest safety levels, the NeuroSIRS-Study will be performed at the Phase I trial unit of the University Hospital of Bonn with a medical emergency team in reach within few minutes.

Only healthy, Caucasian men are included into the trial, unrepresentative of the heterogenous surgical population [65]. It has been demonstrated that LPS-induced inflammatory responses vary between individuals of African vs. European ancestry with a lower inflammatory response in those of African descent [66]. Furthermore, specific differences in the (neuro)-immune and neuroendocrine inflammatory response between men and women have been described. To exclude potential intersexual and interracial differences in immunologic and endocrine response to LPS challenge [67, 68], we accepted this flaw in study design, owing to the exploratory nature of the trial. Of note, due to endotoxin tolerance persisting for an unknown time period, participants who previously underwent endotoxin challenge will be excluded from the study [34].

During the trial, the SmartPill® is used to investigate intestinal motility during SIRS. To generate valid data on specific transit times, changes in gastrointestinal pH measured by the capsule are necessary. It should be noted that pH is highly influenced by VN activation through regulation of secretion of hydrochloric acid [69]. While transit from the stomach to the duodenum is usually well visible, passing of Bauhin’s valve might be hard to detect due to only minimal physiologic changes of 1–2 points in pH value. This would result in difficulties in read out of small bowel and large bowel transit times. However, all other parameters (gastric emptying, whole gut transit, peristaltic activity) measured by the SmartPill® can be analyzed without any issues.

During taVNS a tingling sensation at the outer ear is described, which is not felt during sham stimulation, leading to a potential unblinding during the trial. This problem will be solved during participant education, mentioning that certain sensations might be felt intermediately but are not mandatory. Furthermore, randomization, setting, and placement of the electrode are performed by personnel not further involved in participant care or data extraction/analysis.

Finally, we believe that despite the limitations, the LPS challenge is a suitable and well-controlled approach to mimic postoperative SIRS to ensure reproducible results that allows investigation of the immune modulatory actions of taVNS in humans under inflammatory conditions.

In conclusion, the present study aims to give insight into use of taVNS in humans in a standardized and reproducible model of systemic inflammation. To our knowledge, this is the first study investigating activation of the CAIP using taVNS to potentially reduce symptoms of SIRS and alleviate intestinal failure as a motor of organ failure in a human model. By this pilot trial, we aim to provide a solid basis for further investigations of taVNS as a prophylactic tool in postoperative systemic inflammation and its associated organ failures.

## Data Availability

Study data will be managed confidentially and anonymously. All investigators have unlimited access to the gathered data set without any contractual agreements.
